# U-Shaped Obstacle Avoidance for a Bionic Robotic Fish: A Virtual Sentinel Obstacle Strategy Based on the Artificial Potential Field Method

**DOI:** 10.3390/s26154990

**Published:** 2026-08-06

**Authors:** Yijin Tong, Zhenping Wan, Ruolin Wang, Pengxi Guan, Xiangyu Hu, Qingya Dai

**Affiliations:** School of Mechanical and Automotive Engineering, South China University of Technology, Guangzhou 510641, China; 202421001520@mail.scut.edu.cn (Y.T.); 202521001476@mail.scut.edu.cn (R.W.); meguanpx@mail.scut.edu.cn (P.G.); 202321002312@mail.scut.edu.cn (X.H.); 202520100136@mail.scut.edu.cn (Q.D.)

**Keywords:** bionic robotic fish, U-shaped obstacle, virtual sentinel obstacle, artificial potential field method, fuzzy control

## Abstract

Reliable obstacle avoidance is essential for bionic robotic fish operating in complex underwater environments. However, when a robotic fish performs depth-keeping cruising near U-shaped obstacles, the traditional artificial potential field (APF) method is prone to local minima, which can cause the vehicle to become trapped and lead to obstacle avoidance failure. To address this problem, this paper proposes a virtual sentinel obstacle strategy based on the APF method. Virtual sentinel obstacles are deployed near the entrance of U-shaped obstacles, and corresponding deployment rules are formulated to prevent the robotic fish from entering the local minimum region. To further improve path planning performance, a two-stage fuzzy controller is developed to adjust heading rotation and cruising step size. The proposed method is evaluated through numerical simulations and physical experiments using a self-developed bionic robotic fish prototype. The results show that the virtual sentinel obstacle strategy prevents entrapment around the tested U-shaped obstacles, while fuzzy control shortens the path and improves smoothness. The physical experiments further verify the feasibility of the proposed strategy in a two-dimensional underwater obstacle avoidance scenario. These results indicate that combining virtual sentinel obstacles with APF-based planning provides a feasible approach for U-shaped obstacle avoidance by bionic robotic fish.

## 1. Introduction

Bionic robotic fish combine concealment, maneuverability, and ecological compatibility, making them promising platforms for applications such as underwater equipment inspection and oil and gas infrastructure monitoring [1,2]. During depth-keeping cruising in complex environments, autonomous task execution requires reliable two-dimensional path planning and obstacle avoidance. Concave obstacles, particularly U-shaped obstacles, can guide a robotic fish into a trapping region from which it cannot readily escape, interrupting the mission and potentially requiring manual intervention. Efficient and reliable obstacle avoidance planning for such environments is therefore important for the practical deployment of bionic robotic fish.

A range of methods has been developed for two-dimensional obstacle avoidance by underwater robots. Classical planners such as A* [3] and rapidly exploring random trees (RRTs) [4] can generate global paths, but their paths may require further smoothing and their search cost can increase in complex environments. Direct sensor-based control provides a reactive alternative [5], whereas recent studies have applied water wave optimization [6], differential evolution [7], beetle swarm optimization [8], event-triggered soft actor–critic learning [9], and salp swarm optimization [10].

Related marine robotics studies have also considered integrated motion planning and control for unmanned surface vessels [11], path planning with moving obstacles under environmental uncertainty [12], and fuzzy consensus control for coordinated multi-ship steering [13]. In this context, fuzzy control offers a rule-based way to convert measured states into motion commands. Its input–output mapping can be generated offline and stored as lookup tables, avoiding online training or population-based search. These studies demonstrate the broader value of computationally efficient planning and fuzzy control structures in marine robotic systems, although they do not directly address APF entrapment around static U-shaped obstacles.

The artificial potential field (APF) method is attractive for underwater obstacle avoidance because it requires relatively little computation and naturally generates smooth motion commands. Its main limitation is susceptibility to local minima, which can make the target unreachable in concave environments such as those containing U-shaped obstacles. Existing remedies generally modify either the guidance mechanism or the potential field model. Zhang et al. introduced virtual goal points to guide a mobile robot around linear obstacles [14]. Xie et al. generated a virtual water flow after detecting that a robot had become trapped [15], while Liu et al. combined RRT with APF and used RRT-generated subgoals to guide an autonomous underwater vehicle out of local minima [16]. Other studies modified the potential field formulation itself: Ge et al. incorporated dispersion, directional consistency, and regional differences into a potential field for multiple autonomous underwater vehicles [17], and Xing et al. fused gyroscope and rangefinder data to improve the potential field function [18]. Most of these methods respond to an emerging or detected trapping condition by introducing additional guidance, escape actions, or field adjustments. Around U-shaped obstacles, such post-trapping processing can require extra online detection, escape search, or replanning, increasing the computational burden on the underwater platform.

To address this limitation, this study places a virtual sentinel obstacle outside the opening of a U-shaped obstacle to reshape the local potential field before the robotic fish enters the trapping region. A two-stage fuzzy controller then adjusts the heading and cruising step size of the virtual sentinel obstacle APF. The strategy is evaluated through numerical simulations and experiments on a self-developed bionic robotic fish platform.

## 2. Virtual Sentinel Obstacle Avoidance Strategy

### 2.1. Simplification of the U-Shaped Obstacle in the APF

**Figure 1:**
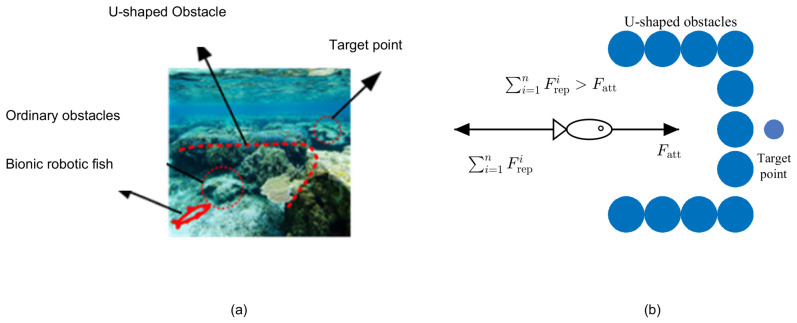
Physical and APF representations of a U-shaped obstacle. (**a**) Physical scene, with red markings indicating the obstacle boundary, robotic fish, and target. (**b**) APF model, where the dark- and light-blue circles denote obstacle elements and the target, respectively.

Obstacles in underwater environments can have irregular geometries, including the U-shaped obstacle shown in Figure 1a. For APF analysis, the U-shaped boundary is represented by a series of circular obstacles, and the target is represented by a point (Figure 1b). The bionic robotic fish is approximated by a point at its center of mass.

In the APF, the target exerts an attractive force $F_{\mathrm{att}}$ directed from the current fish position toward the target. Obstacle *i* exerts a repulsive force $F_{\mathrm{rep}}^{i}$ directed from that obstacle toward the current position. For *n* obstacles, the total repulsive force is

(1) $$F_{\mathrm{rep}}^{\mathrm{total}}=\sum_{i=1}^{n}F_{\mathrm{rep}}^{i},\;n\geq 1.$$

The resultant force acting on the bionic robotic fish is

(2) $$F=F_{\mathrm{att}}+F_{\mathrm{rep}}^{\mathrm{total}}.$$

Near or inside the U-shaped obstacle, the attractive and total repulsive forces can oppose and balance each other (Figure 1b). The resultant force then approaches zero before the target is reached, producing a local minimum that can trap the bionic robotic fish.

### 2.2. Virtual Sentinel Obstacle Strategy

**Figure 2:**
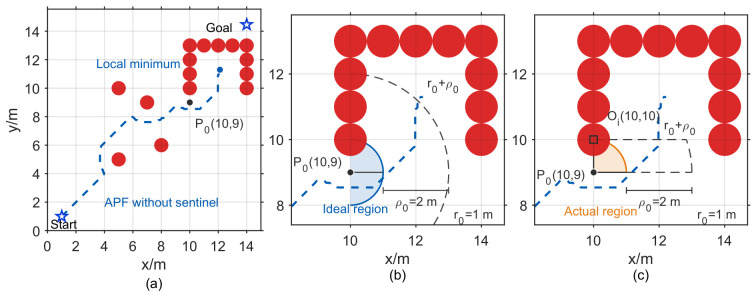
Virtual sentinel obstacle placement and feasible deployment regions. (**a**) APF trajectory without a virtual sentinel obstacle. (**b**) Ideal candidate region. (**c**) Actual feasible region. Red circles denote obstacles.

Most escape-oriented APF methods respond after the robot has entered a concave region or stagnation has been detected. The virtual sentinel obstacle strategy instead modifies the resultant force near the entrance of a U-shaped obstacle before the robotic fish reaches the local minimum region. Let the start and target points be denoted by *start* and *goal*, respectively. The point $P_{0}=\left(x_{0},y_{0}\right)$ is the entrance point at which the original APF trajectory begins to enter the U-shaped obstacle. Without a virtual sentinel obstacle, the fish proceeds from $P_{0}$ into the cavity and becomes trapped at $\left(x_{1},y_{1}\right)$, as illustrated in Figure 2a.

Let $s=(x_{s},y_{s})$ denote the center of the virtual sentinel obstacle. The deployment rule is constructed in two stages. First, an ideal candidate region is defined within a distance $r_{0}$ of $P_{0}$ on the entrance-side:

(3) $${\left(x_{s}-x_{0}\right)}^{2}+{\left(y_{s}-y_{0}\right)}^{2}\leq r_{0}^{2},\;x_{s}\geq x_{0}.$$

Here, $r_{0}$ is the maximum admissible distance between $P_{0}$ and the center of the virtual sentinel obstacle, and $\rho_{0}$ is its repulsive influence radius. The distance constraint keeps the center close enough to intercept the incoming APF trajectory. The inequality $x_{s}\geq x_{0}$ selects the entrance-side half-neighborhood represented by the solid boundary in Figure 2b. If every candidate center has the same influence radius $\rho_{0}$, the outer envelope of the candidate influence disks is bounded by

(4) $${\left(q_{x}-x_{0}\right)}^{2}+{\left(q_{y}-y_{0}\right)}^{2}\leq {\left(r_{0}+\rho_{0}\right)}^{2},\;q_{x}\geq x_{0},$$

where $q=(q_{x},q_{y})$ is an arbitrary point in the entrance-side influence envelope. The dashed boundary in Figure 2b denotes the corresponding $r_{0}+\rho_{0}$ envelope. For Equations (3)–(6), the local coordinate frame is aligned so that the positive *x*-direction points from $P_{0}$ toward the obstacle interior; a rotated obstacle can be treated by applying the same constraints in its local frame. Let $O_{i}=\left(x_{i},y_{i}\right)$ denote the nearest obstacle point on the upper entrance edge. To keep the virtual sentinel obstacle outside the cavity, its center is restricted to the entrance-side region bounded by $P_{0}$ and $O_{i}$. The feasible center set is therefore

(5) $$D=\left\{s:{\left(x_{s}-x_{0}\right)}^{2}+{\left(y_{s}-y_{0}\right)}^{2}\leq r_{0}^{2},\;x_{s}\geq x_{i},\;y_{0}\leq y_{s}\leq y_{i}\right\}.$$

Equation (5) defines where the center of the virtual sentinel obstacle may be placed, and the resulting feasible region is shown in Figure 2c. For a selected center s∈D, the virtual sentinel obstacle acts on points within $\rho_{0}$. The influence region associated with all admissible centers is therefore

(6) $$I\left(D,\rho_{0}\right)=\underset{s\in D}{⋃}\left\{q:∥q-s∥\leq \rho_{0}\right\}\subseteq \left\{q:∥q-p_{0}∥\leq r_{0}+\rho_{0}\right\},$$

where $p_{0}=\left(x_{0},y_{0}\right)$ is the position vector of $P_{0}$, D is the feasible center set defined in Equation (5), and $I(D,\rho_{0})$ is the union of the influence disks generated by all centers in D. Equations (5) and (6) therefore have different roles. Equation (5) constrains the center position, whereas Equation (6) describes the region in which an admissible virtual sentinel obstacle can modify the APF trajectory.

The force requirement can be obtained by projecting the resultant force onto the outward normal of the local entrance boundary through $P_{0}$. Let $n_{\mathrm{out}}$ be the unit normal directed from this boundary toward the free-space side, and let $F_{0}\left(P_{0}\right)$ be the resultant APF force at $P_{0}$ without the virtual sentinel obstacle. In the simulation, the virtual sentinel obstacle uses the same repulsive coefficient $k_{\mathrm{rep}}$ as the real obstacles; no additional gain is introduced. Dividing all force terms by this common coefficient gives ${\tilde{F}}_{0}=F_{0}/k_{\mathrm{rep}}$. The normalized inward component is

(7) $${\tilde{F}}_{\mathrm{in}}=\max\left(0,-{\tilde{F}}_{0}\left(P_{0}\right)\cdot n_{\mathrm{out}}\right).$$

For a candidate center s, define $d_{0}=∥p_{0}-s∥$ and $u_{s}=\left(p_{0}-s\right)/d_{0}$. The corresponding normalized force generated by the virtual sentinel obstacle is

(8) $${\tilde{F}}_{s}\left(P_{0}\right)=\phi \left(d_{0},\rho_{0}\right)u_{s},\;\phi \left(d_{0},\rho_{0}\right)=\left\{\begin{array}{cc} \left(\frac{1}{d_{0}}-\frac{1}{\rho_{0}}\right)\frac{1}{d_{0}^{2}}, & d_{0}<\rho_{0},\\ 0, & d_{0}\geq \rho_{0}. \end{array}\right.$$

Let $c=u_{s}\cdot n_{\mathrm{out}}$. A candidate center can oppose entry only when c>0. The virtual sentinel obstacle prevents an inward resultant force at $P_{0}$ when

(9) $$d_{0}<\rho_{0},\;c>0,\;\phi \left(d_{0},\rho_{0}\right)c\geq {\tilde{F}}_{\mathrm{in}},$$

because Equation (9) gives $\left({\tilde{F}}_{0}+{\tilde{F}}_{s}\right)\cdot n_{\mathrm{out}}\geq 0$ at the entrance. This projection condition provides the mechanical basis for the geometric rules. Equation (5) supplies candidate centers close to and outside the entrance. Equation (6) requires the incoming trajectory to lie within the influence region of the virtual sentinel obstacle. Equation (9) rejects candidates whose repulsive direction or magnitude cannot counter the inward APF component. For the simulated placement $P_{0}=\left(10,9\right)$ and s=(11,9), the free-space normal is $n_{\mathrm{out}}=\left(-1,0\right)$, giving $d_{0}=1$, c=1, ϕ=0.5, and ${\tilde{F}}_{\mathrm{in}}=0.04$. Thus, the condition 0.5≥0.04 is satisfied. The implemented planner selects the lowest-potential point from eight neighboring candidates. Equation (9) is therefore a local directional consistency condition for the underlying continuous potential field rather than a proof of global convergence. The resulting discrete trajectory is verified separately by simulation. The parameter $r_{0}$ controls how far the center may be placed from $P_{0}$, whereas $\rho_{0}$ controls both whether the entrance is reached and the repulsive magnitude there. If either parameter is too small, the virtual sentinel obstacle may fail to intercept the trajectory. If either parameter is excessive, the path may become unnecessarily conservative or a new equilibrium may be introduced.

### 2.3. Simulation and Parameter Sensitivity Analysis

The simulated trajectories were evaluated using path length, minimum obstacle clearance, and a smoothness index. For a path containing *n* points $p_{i}=\left(x_{i},y_{i}\right)$, the path length is

(10) $$L=\sum_{i=1}^{n-1}{∥p_{i+1}-p_{i}∥}_{2}.$$

For circular obstacle *j* with center $o_{j}$ and radius $R_{j}$, the minimum clearance is

(11) $$d_{\min}=\min_{i,j}\left({∥p_{i}-o_{j}∥}_{2}-R_{j}\right).$$

Path smoothness is measured by the accumulated absolute change in heading:

(12) $$S=\sum_{i=1}^{n-2}\left|{\mathrm{wrap}}_{\left[-\pi ,\pi \right)}\;\left(\theta_{i+1}-\theta_{i}\right)\right|,\;\theta_{i}=atan2\;\left(y_{i+1}-y_{i},x_{i+1}-x_{i}\right).$$

A smaller *S* indicates fewer or weaker heading changes. Successful avoidance requires arrival at the target together with positive minimum clearance.

MATLAB R2023a simulations were conducted in a fixed U-shaped obstacle environment. The four ordinary obstacles are centered at (5,5), (5,10), (8,6), and (7,9), and the U-shaped obstacle is formed by circular obstacles centered at (10,10), (10,11), (10,12), (10,13), (11,13), (12,13), (13,13), (14,13), (14,12), (14,11), and (14,10). The start and target points are (1,1) and (14,15), respectively. Without a virtual sentinel obstacle, the conventional APF did not reach the target and terminated with a final target distance of 4.155 m after the maximum path length of 200 m.

The joint sensitivity of the deployment radius and influence radius was then evaluated using $r_{0}=\left\{0.5,1.0,1.5,2.0\right\}$ m and $\rho_{0}=\left\{1.0,1.5,2.0,2.5\right\}$ m. Here, $P_{0}=\left(10,9\right)$ and $O_{i}=\left(10,10\right)$. For each $r_{0}$, the representative boundary center $s=(x_{0}+r_{0},y_{0})$ was selected from Equation (5); thus, $d_{0}=r_{0}$ and c=1. Equation (9) was first used as a local screening condition, whereas successful avoidance was determined from the complete discrete trajectory by requiring target arrival and positive clearance from all real obstacles.

**Figure 3:**
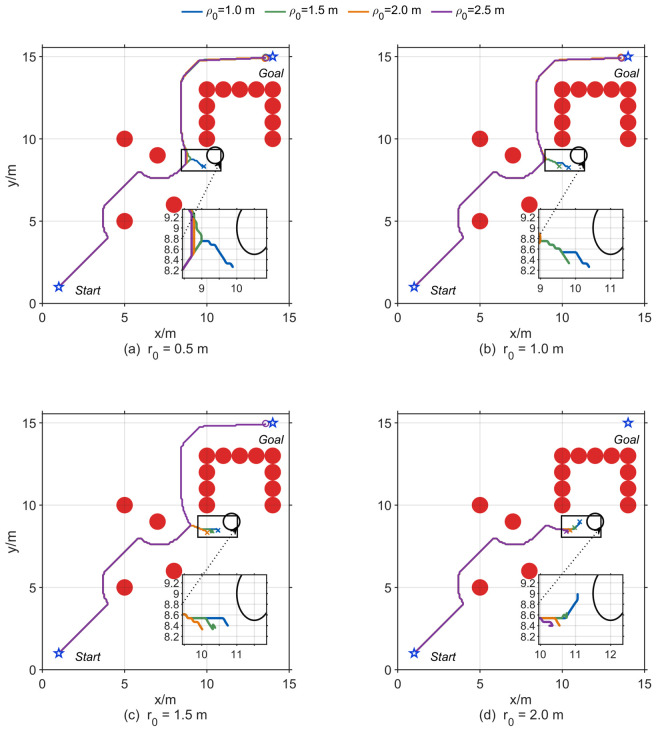
Path sensitivity to $r_{0}$ and $\rho_{0}$ for (**a**) $r_{0}=0.5$ m, (**b**) $r_{0}=1.0$ m, (**c**) $r_{0}=1.5$ m, and (**d**) $r_{0}=2.0$ m. Red circles indicate obstacles; colored lines show trajectories for the $\rho_{0}$ values in the legend.

As shown in Figure 3, 9 of the 16 parameter combinations satisfied the local condition in Equation (9), but only 6 reached the target. The combinations $\left(r_{0},\rho_{0}\right)=\left(0.5,1.0\right)$, (1.0,1.5), and (1.5,2.0) m passed the local screening but failed in the complete discrete trajectory, confirming that Equation (9) is not a global convergence condition. Successful avoidance required $\rho_{0}\geq 1.5$ m for $r_{0}=0.5$ m, $\rho_{0}\geq 2.0$ m for $r_{0}=1.0$ m, and $\rho_{0}=2.5$ m for $r_{0}=1.5$ m. None of the tested influence radii succeeded when $r_{0}=2.0$ m. This trend agrees with the geometric interpretation that a virtual sentinel obstacle placed farther from the entrance requires a larger influence radius. The selected setting $r_{0}=1.0$ m and $\rho_{0}=2.0$ m places its center at (11,9), satisfies 0.5≥0.04 in Equation (9), reaches the target with a path length of 23.800 m, and maintains a minimum clearance of 0.811 m.

## 3. Fuzzy Control Optimization

The virtual sentinel obstacle can increase path updates near ordinary obstacles. A two-stage Mamdani fuzzy controller [19] is therefore used to coordinate heading rotation and cruising step size while retaining a low-cost lookup-table implementation.

### 3.1. Design of Fuzzy Controller

**Figure 4:**
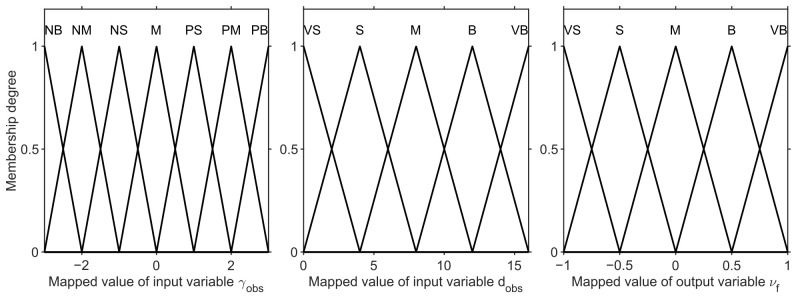
Triangular membership functions of the two-stage fuzzy controller: $\gamma_{\mathrm{obs}}$; $d_{\mathrm{obs}}$; $\nu_{f}$.

The first stage maps obstacle distance $d_{\mathrm{obs}}$ and the angle $\gamma_{\mathrm{obs}}$ between the repulsive and resultant forces to heading rotation $\gamma_{\mathrm{rot}}$. The second stage maps target distance $d_{g}$ and $\gamma_{\mathrm{rot}}$ to cruising speed variable $\nu_{f}$. Directional variables use seven linguistic states {NB, NM, NS, M, PS, PM, PB}, whereas distance and speed variables use five states {VS, S, M, B, VB}. Angles in [−π,π] are mapped to [−3,3], distances are bounded to [0,16], and $\nu_{f}\in \left[-1,1\right]$ is converted to the step-size scale $(\nu_{f}+1)/2$. Triangular membership functions were selected for their piecewise-linear evaluation and continuous overlap (Figure 4).

Table 1 and Table 2 give the first- and second-stage rule bases.

The rules impose three constraints: $\gamma_{\mathrm{rot}}$ follows the avoidance direction, its magnitude increases for closer obstacles or stronger angular conflict, and the cruising step decreases near the target or during large rotations. Mamdani inference uses minimum implication, maximum aggregation, unit rule weights, and centroid defuzzification:

(13) $$Q_{0}=\frac{\int_{\Omega }q\mu \left(q\right)\;dq}{\int_{\Omega }\mu \left(q\right)\;dq},$$

where $Q_{0}$ is the defuzzified output, *q* is the output-domain variable, μ(q) is the aggregated membership degree, and Ω is the output universe. The resulting input–output surfaces are discretized offline; online control requires only input scaling and lookup of the heading and step-size commands.

### 3.2. Sensitivity of the Fuzzy Controller

Under the same U-shaped environment and virtual sentinel obstacle position, the sensitivity analysis varied the controller stages, membership shape, defuzzification method, and rule weight. APF with the virtual sentinel obstacle but without fuzzy adjustment was included as the reference. Path length, clearance, and smoothness were calculated using Equations (10)–(12) (Table 3).

The sensitivity comparison showed that path quality depended on controller tuning. Mean-of-maximum gave the shortest path (23.290 m), whereas the triangular–centroid controller gave a comparable path (23.371 m) and the lowest smoothness index among the tested defuzzification methods (10.996 rad). The single-stage, Gaussian, bisector, and rule-weight-0.6 settings produced substantially longer or more oscillatory paths. The triangular–centroid structure was therefore retained as the best path-length–smoothness compromise in the tested environment.

### 3.3. Simulation Analysis

**Figure 5:**
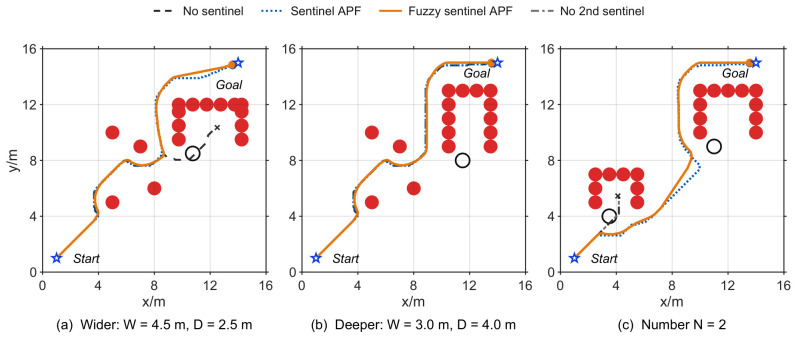
Obstacle avoidance paths under different U-shaped obstacle geometries: (**a**) wider and shallower U-shaped obstacle; (**b**) narrower and deeper U-shaped obstacle; (**c**) two U-shaped obstacles. Red circles denote obstacles.

To complement the controller tuning analysis under the fixed U-shaped environment in Section 3.2, additional simulations varied the U-shaped geometry and virtual sentinel obstacle configuration. Here, *W* denotes the obstacle-opening width and *D* denotes the concavity depth. Figure 5a uses a wider and shallower opening (W=4.5 m, D=2.5 m) with $\rho_{0}=3.0$ m. Figure 5b uses a narrower and deeper concavity (W=3.0 m, D=4.0 m) with $\rho_{0}=2.0$ m. Figure 5c contains two U-shaped obstacles and uses one virtual sentinel obstacle for each U-shaped obstacle, with $\rho_{0}=2.5$ m. A staged path with the second virtual sentinel obstacle omitted isolates the effect of the multi-sentinel configuration.

The geometric-layout comparison further quantified the effect of fuzzy adjustment. In the wider and shallower case, fuzzy control reduced the path length from 23.200 to 22.519 m and the smoothness index from 52.622 to 12.763 rad. In the narrower and deeper case, it reduced the path length from 23.100 to 22.665 m and the smoothness index from 29.060 to 9.032 rad. Its effect remained evident in the two-U environment, reducing the path length from 24.200 to 23.002 m and the smoothness index from 29.845 to 8.247 rad. Thus, the strategy preserved its obstacle avoidance capability under geometric changes and provided greater optimization in the more complex layout.

## 4. Experiments and Results Analysis

### 4.1. Prototype of Bionic Robotic Fish and Experimental Platform

**Figure 6:**
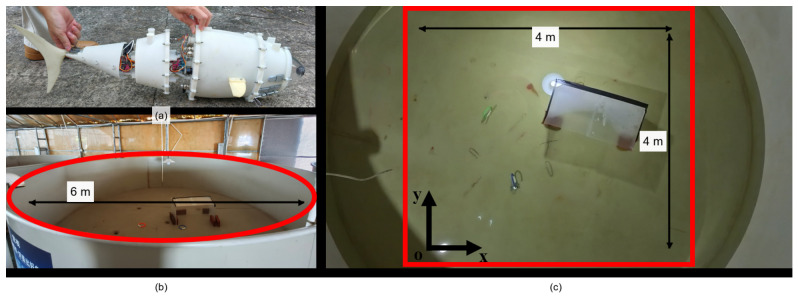
Bionic robotic fish and experimental platform: (**a**) robotic fish prototype; (**b**) circular pool with a diameter of 6 m; (**c**) 4m×4m experimental area.

A bionic robotic fish was developed as the experimental prototype. An STM32F407VET6 microcontroller executes the onboard control commands at an update frequency of 50 Hz, and the prescribed oscillation frequency is 2 Hz. A shore-based computer transmits the start command through wireless communication. The experiments were conducted in a circular pool with a diameter of 6 m (Figure 6), which provided a larger test space than the tank used in the original submission. A 4m×4m square near the center of the pool defined the experimental area and the world coordinate domain. Centering the motion layout increased its distance from the pool wall and was intended to reduce wall-induced interference. An overhead camera recorded the motion for subsequent trajectory digitization. The dimension line in Figure 6b marks the 6 m pool diameter, while the horizontal and vertical dimension lines in Figure 6c mark the 4m×4m experimental area.

The origin *O* was placed at the lower-left corner of the square experimental area in Figure 6c. The positive *x*-axis points to the right, and the positive *y*-axis points upward, defining the world coordinate system used to describe the planar motion of the robotic fish.

The physical obstacle positions were expressed in this coordinate system. The U-shaped obstacle was 1.4 m long and 0.6 m wide. Table 4 lists its center and the ordinary obstacle coordinates for each arrangement. The virtual sentinel obstacle was deployed according to Section 2.2.

### 4.2. Experimental Results

**Figure 7:**
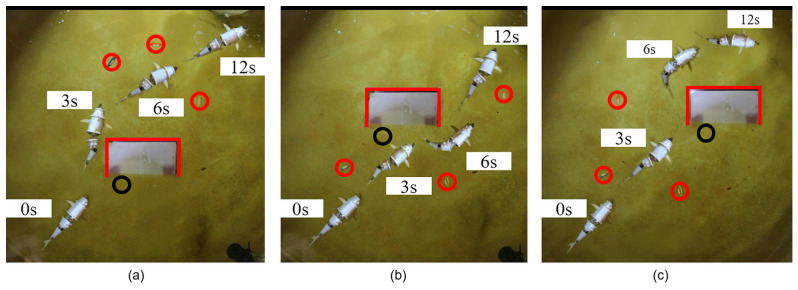
Representative obstacle avoidance sequences under (**a**) arrangement 1, (**b**) arrangement 2, and (**c**) arrangement 3. Labels show fish positions at 0, 3, 6, and 12 s. Red circles and red U-shaped outlines denote obstacles; black circles denote virtual sentinel obstacles.

The virtual sentinel obstacle strategy was evaluated under the three obstacle arrangements in Table 4. Each method was evaluated in five runs under each arrangement. The 50 Hz control update, 2 Hz oscillation, and 1 s camera-based position sampling served different purposes: the first updated the motion command, the second prescribed the oscillatory motion, and the third generated the recorded trajectory. The overhead camera images yielded a discrete time-ordered set of planar localization points for each run. Figure 7a–c superimposes representative fish positions at 0, 3, 6, and 12 s. Red circles denote ordinary obstacles, and the red U-shaped boundary denotes the concave obstacle. The representative sequences show the fish passing the obstacle region without becoming trapped inside the U-shaped obstacle.

**Figure 8:**
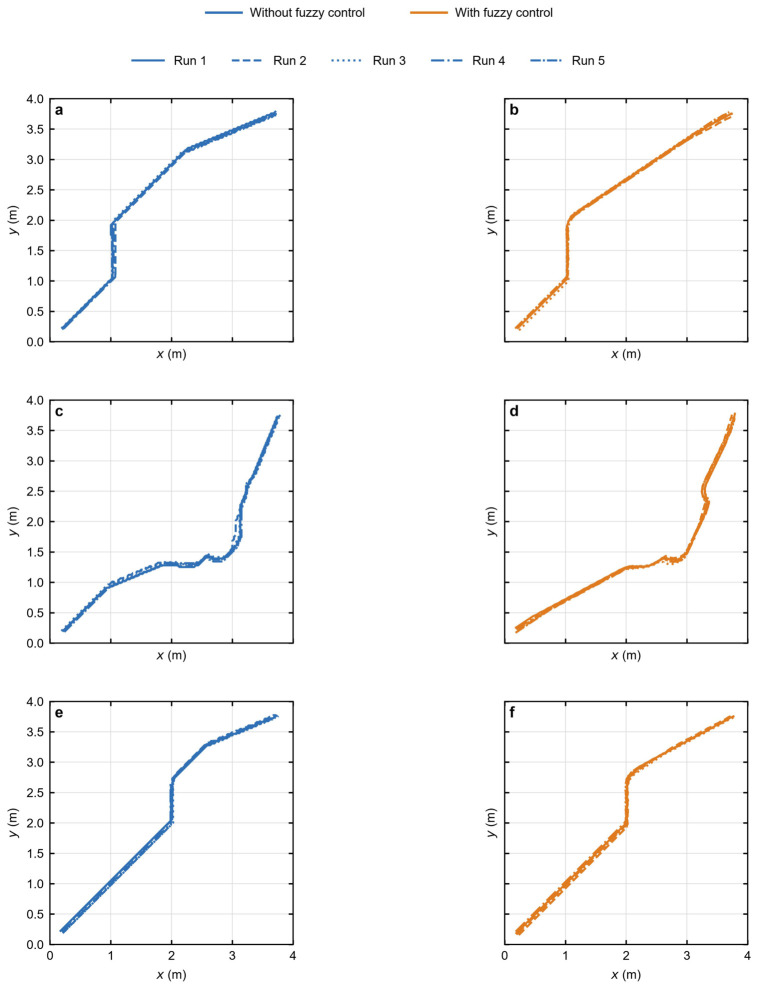
Repeated experimental trajectories under three obstacle arrangements: (**a**,**b**) arrangement 1; (**c**,**d**) arrangement 2; and (**e**,**f**) arrangement 3. Panels (**a**,**c**,**e**) show results without fuzzy control, whereas panels (**b**,**d**,**f**) show results with fuzzy control. Blue and orange lines distinguish the two methods, respectively; different line styles denote Runs 1–5.

After each run, the *x*- and *y*-coordinate sequences were linearly interpolated with respect to time. The interpolated coordinates were connected chronologically to reconstruct a continuous piecewise-linear trajectory. The start and target positions were (0.20,0.20) and (3.80,3.80) m, respectively. Figure 8 shows the five reconstructed trajectories for each method and arrangement. The average path length and accumulated heading change for each method–arrangement group were calculated from these five runs.

**Figure 9:**
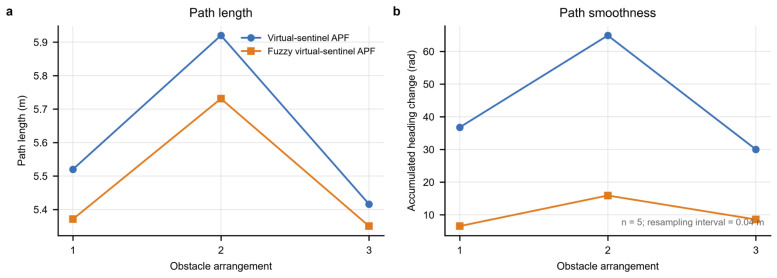
Experimental comparison of path length and accumulated heading change: (**a**) average path length across the three obstacle arrangements; (**b**) average accumulated heading change across the three obstacle arrangements. Blue circles denote the virtual-sentinel APF, and orange squares denote the fuzzy virtual-sentinel APF. Each point represents the mean of five runs.

To remove the effects of the original 1 s temporal sampling interval and local variations in swimming speed, each interpolated trajectory was resampled at a uniform arc-length interval of 0.04 m. Path length was calculated from the resampled coordinates using Equation (10). For each pair of consecutive resampled points, the segment heading was obtained using the atan2 definition in Equation (12). The smoothness index was then calculated by summing the absolute wrapped heading changes between adjacent segments. Thus, the complete experimental processing chain comprised 1 s position sampling, time-domain linear interpolation, 0.04 m arc-length resampling, and equation-based metric calculation. Figure 9 reports the average values across five runs. For arrangements 1–3, fuzzy control reduced the average path length from 5.520 to 5.371 m, from 5.920 to 5.731 m, and from 5.416 to 5.350 m, respectively. The corresponding average smoothness indices decreased from 36.757 to 6.532 rad, from 64.874 to 15.900 rad, and from 30.002 to 8.564 rad. Across the three tested arrangements, the virtual sentinel obstacle prevented entry into the U-shaped local minimum region, whereas the fuzzy controller reduced unnecessary detours and abrupt heading changes. The combined method retains the low computational complexity and online interpretability of APF-based control while improving path efficiency and motion continuity for a resource-constrained robotic fish.

## 5. Conclusions

This study proposes a virtual sentinel obstacle APF strategy and a two-stage fuzzy controller for robotic fish avoidance of U-shaped obstacles. Simulations and pool experiments show that the virtual sentinel obstacle redirects the fish before it enters the concave local minimum region. After the experimental trajectories were resampled at a common spatial interval, fuzzy control shortened the path and reduced accumulated heading change in all three tested arrangements. By preventing entrapment rather than initiating an escape after entrapment, the virtual sentinel obstacle preserves the simple force-based structure of the conventional APF. The fuzzy controller further adjusts heading and step size online without iterative global optimization or offline training. Together, these features provide a computationally lightweight, interpretable, and experimentally feasible solution for real-time obstacle avoidance on robotic fish with limited onboard resources.

The present method remains limited to static U-shaped obstacles during two-dimensional, depth-keeping motion. The experiments were performed in a large pool with the motion layout positioned near its center to reduce wall-induced interference, but the results should not be extrapolated to environments with water currents, dynamic obstacles, or severe visual occlusion. Virtual sentinel obstacle placement assumes reliable detection of the obstacle entrance and geometry; detection error or occlusion could therefore produce an unsuitable placement. Multiple closely spaced U-shaped obstacles may produce overlapping repulsive fields and require coordinated deployment. In addition, an unsuitable center position or influence radius could create a new local minimum or an unnecessary detour.

Future work will investigate localization calibration, onboard timing measurements, uncertainty-aware perception, adaptive placement of multiple virtual sentinel obstacles, current disturbances, dynamic obstacles, and extension to three-dimensional motion.

## Figures and Tables

**Table 1:** First-stage fuzzy control rule base for $\gamma_{\mathrm{rot}}$.

	$\gamma_{\mathrm{obs}}$	NB	NM	NS	M	PS	PM	PB
$d_{\mathrm{obs}}$								
VS		NB	NB	NM	NS	PM	PB	PB
S		NB	NM	NM	NS	PM	PM	PB
M		NM	NM	NS	M	PS	PM	PM
B		NS	NS	M	PS	M	PS	PS
VB		M	M	M	PS	M	M	M

*Note:* The column variable is $\gamma_{\mathrm{obs}}$, the row variable is $d_{\mathrm{obs}}$, and each intersection gives the output linguistic value of $\gamma_{\mathrm{rot}}$.

**Table 2:** Second-stage fuzzy control rule base for $\nu_{f}$.

	$\gamma_{\mathrm{rot}}$	NB	NM	NS	M	PS	PM	PB
$d_{g}$								
VS		VS	VS	VS	VS	VS	VS	VS
S		VS	S	S	S	S	VS	PB
M		S	S	M	M	M	S	S
B		S	M	B	B	B	M	S
VB		M	B	VB	VB	VB	B	M

*Note:* The column variable is $\gamma_{\mathrm{rot}}$, the row variable is $d_{g}$, and each intersection gives the output linguistic value of $\nu_{f}$.

**Table 3:** Sensitivity of the fuzzy controller design choices.

Controller	MF	Defuzz.	Weight	Path/m	$d_{\min}$/m	*S*/rad
No fuzzy control	–	–	–	23.800	0.811	36.128
Single-stage	Triangular	Centroid	1.0	53.750	0.588	611.825
Two-stage	Triangular	Centroid	1.0	23.371	0.696	10.996
Two-stage	Gaussian	Centroid	1.0	49.719	0.589	653.059
Two-stage	Triangular	Bisector	1.0	51.728	0.591	666.803
Two-stage	Triangular	Mean of max.	1.0	23.290	0.694	15.708
Two-stage	Triangular	Centroid	0.8	23.391	0.708	10.996
Two-stage	Triangular	Centroid	0.6	48.633	0.590	616.930

MF: membership function; Defuzz.: defuzzification method.

**Table 4:** Obstacle dimensions and coordinates for the experimental arrangements.

Arrangement	U-Shaped Obstacle Size	U-Shaped Obstacle Center	Ordinary Obstacle Centers
1	1.4m×0.6m	(2.00,1.50)	(2.25,3.65); (1.60,3.40); (3.00,2.40)
2	1.4m×0.6m	(2.00,2.25)	(1.20,1.75); (2.75,1.60); (3.50,2.50)
3	1.4m×0.6m	(3.00,2.25)	(1.00,1.65); (2.10,1.10); (1.20,2.45)

Coordinates are reported in metres as (x,y). The obstacle size is given as length × width.

## Data Availability

The data used to support the findings of this study are available within the article.

## Abbreviations

The following abbreviations are used in this manuscript:

APF	Artificial potential field
RRT	Rapidly exploring random tree
AUV	Autonomous underwater vehicle
